# β-Mangostin Alleviates Renal Tubulointerstitial Fibrosis via the TGF-β1/JNK Signaling Pathway

**DOI:** 10.3390/cells13201701

**Published:** 2024-10-14

**Authors:** Po-Yu Huang, Ying-Hsu Juan, Tung-Wei Hung, Yuan-Pei Tsai, Yi-Hsuan Ting, Chu-Che Lee, Jen-Pi Tsai, Yi-Hsien Hsieh

**Affiliations:** 1 Institute of Medical Sciences, Tzu Chi University, Hualien 970374, Taiwan; poyuhs13628@gmail.com; 2 Division of Nephrology, Department of Internal Medicine, Dalin Tzu Chi Hospital, Buddhist Tzu Chi Medical Foundation, Chiayi 62247, Taiwan; 3 Department of Chinese Medicine, Dalin Tzu Chi Hospital, Buddhist Tzu Chi Medical Foundation, Chiayi 62247, Taiwan; ddjjaa@tcts.seed.net.tw; 4 School of Post-Baccalaureate Chinese Medicine, Tzu Chi University, Hualien 970374, Taiwan; 5 School of Medicine, Chung Shan Medical University, Taichung 40201, Taiwan; a6152000@ms34.hinet.net; 6 Division of Nephrology, Department of Medicine, Chung Shan Medical University Hospital, Taichung 40201, Taiwan; 7 Institute of Medicine, Chung Shan Medical University, Taichung 40201, Taiwan; patty8782@gmail.com (Y.-P.T.); dys0090@gmail.com (Y.-H.T.); 8 Department of Medicine Research, Buddhist Dalin Tzu Chi Hospital, Chiayi 62247, Taiwan; dm731849@tzuchi.com.tw; 9 School of Medicine, Tzu Chi University, Hualien 970374, Taiwan; 10 Department of Medical Research, Chung Shan Medical University Hospital, Taichung 40201, Taiwan

**Keywords:** β-mangostin, renal tubulointerstitial fibrosis, EMT, TGF-β1, Smad2, JNK1/2

## Abstract

The epithelial-to-mesenchymal transition (EMT) plays a key role in the pathogenesis of kidney fibrosis, and kidney fibrosis is associated with an adverse renal prognosis. Beta-mangostin (β-Mag) is a xanthone derivative obtained from mangosteens that is involved in the generation of antifibrotic and anti-oxidation effects. The purpose of this study was to examine the effects of β-Mag on renal tubulointerstitial fibrosis both in vivo and in vitro and the corresponding mechanisms involved. As shown through an in vivo study conducted on a unilateral ureteral obstruction mouse model, oral β-Mag administration, in a dose-dependent manner, caused a lesser degree of tubulointerstitial damage, diminished collagen I fiber deposition, and the depressed expression of fibrotic markers (collagen I, α-SMA) and EMT markers (N-cadherin, Vimentin, Snail, and Slug) in the UUO kidney tissues. The in vitro part of this research revealed that β-Mag, when co-treated with transforming growth factor-β1 (TGF-β1), decreased cell motility and downregulated the EMT (in relation to Vimentin, Snail, and N-cadherin) and phosphoryl-JNK1/2/Smad2/Smad3 expression. Furthermore, β-Mag co-treated with SB (Smad2/3 kinase inhibitor) or SP600125 (JNK kinase inhibitor) significantly inhibited the TGF-β1-associated downstream phosphorylation and activation of JNK1/2-mediated Smad2 targeting the Snail/Vimentin axis. To conclude, β-Mag protects against EMT and kidney fibrotic processes by mediating the TGF-β1/JNK/Smad2 targeting Snail-mediated Vimentin expression and may have therapeutic implications for renal tubulointerstitial fibrosis.

## 1. Introduction

Kidney fibrosis, a typical morphological consequence of chronic kidney failure with various etiologies, is characterized by the disordered repair and healing of the renal parenchyma following an acute or sustained injurious insult [1]. Kidney interstitial fibrogenesis is a complicated process involving plenteous cell types and molecular pathways [2]. With the stimulation of TGF-β and downstream Smad signaling, the renal proximal tubular epithelial cells undergo a partial epithelial-to-mesenchymal transition (EMT) and are transformed into a myofibroblast phenotype [3,4,5]. Activated myofibroblasts derived from the dedifferentiated epithelial cells, pericytes, and perivascular fibroblasts proliferate and enhance extracellular matrix deposition [6,7]. Transcription factors such as Snail, Slug, and Twist, as well as alpha smooth muscle actin (α-SMA), Vimentin, N-cadherin, type I collagen, and fibronectin, constitute the biomarkers representative of mesenchymal differentiation and chronic fibrosis [8,9]. In addition, kidney damage upregulates the c-Jun amino terminal kinase (JNK) pathway, which interacts with TGF-β-dependent profibrotic effects and induces cell apoptosis [10]. Other TGF-β-driven mitogen-activated protein kinases, namely, extracellular signal-regulated kinase 1/2 [ERK1/2, downstream mitogen-activated protein kinase kinase (MEK1/2)], and p38 kinase, are also implicated in the pathomechanism of kidney fibrosis [11]. Other mechanisms contributing to kidney fibrosis are leukocyte infiltration with an induction of inflammation, capillary rarefaction with the generation of a hypoxic milieu, diminished fatty acid oxidation, and epigenetic alterations in myofibroblasts [12]. Kidney fibrosis is associated with the progression of chronic kidney disease and an increased risk of end-stage kidney disease [13].

*Garcinia mangostana* L., bearing the tropical fruit known as a mangosteen, has long been used for therapy, including for infectious diseases, inflammatory conditions, and pyrexia [14]. The pericarp of a mangosteen contains a number of compounds containing xanthone elements, such as α-, β-, and γ-mangostin [15]. Among these molecules, β-mangostin (β-Mag) carries out some critical biologic actions. Regarding anti-inflammatory properties, β-Mag inhibited the nuclear translocation of nuclear factor-κB and suppressed the production of lipopolysaccharide-induced proinflammatory cytokines in cultured macrophage cell lines [16]. Furthermore, β-Mag has antineoplastic effects and retards cancer invasion. β-Mag brought about cytotoxicity against rat glioma cells through the introduction of oxidative stress and the inhibition of phosphatidylinositol-3-kinase phosphorylation and downstream signaling [17]. In MCF-7 breast cancer cell lines, β-Mag upregulated apoptotic enzymes and caused cell cycle arrest [18]. β-Mag decreased the metastatic reserve and proliferation of human cervical tumor cells via the downregulation of JNK2 expression [19]. On the other hand, β-Mag activated ERK and JNK pathways, reducing the invasion of human hepatocellular carcinoma cells [20].

Based on findings from the literature, the antifibrotic effect of β-Mag is not well understood. Since its tissue fibrotic processes are closely associated with inflammation and cell proliferation, we hypothesize that β-Mag has an impact on signaling cascades linked to kidney fibrosis. In the current investigation, we studied the in vitro and in vivo roles of β-Mag in the alleviation and reversion of kidney fibrosis.

## 2. Materials and Methods

### 2.1. Chemicals and Reagents

β-Mag (purity >98%, determined by HPLC) was obtained from Chengdu Efa Bio Limited Company (Chengdu, China). SP600125 (SP) and SB431542 (S-7800) were purchased from LC Labor-atories (Woburn, MA, USA). TGF-β1 (240-B-010) was purchased from Bio-Techne Corporation (Minneapolis, MN, USA). The JNK plasmid was kindly provided by Professor Shun-Fa Yang (Chung Shan Medical University).

### 2.2. The Unilateral Ureteral Obstruction (UUO) Model and Treatment

**Figure 1:**
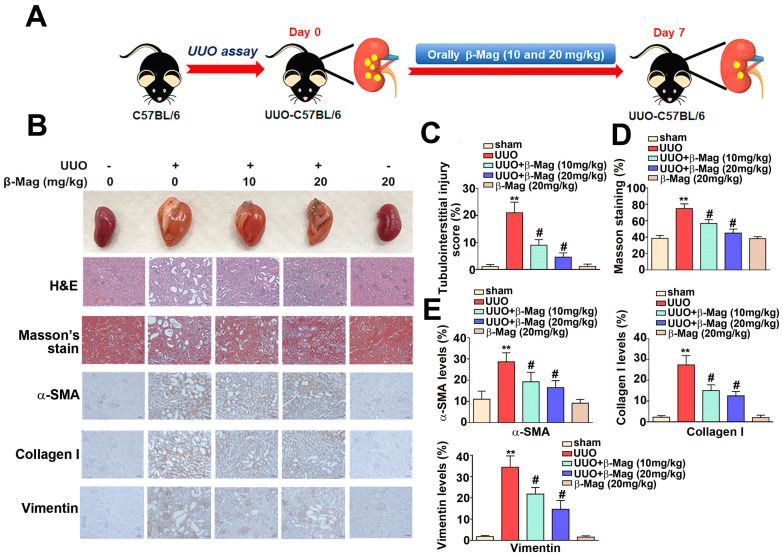
Histopathological examination of kidney tissue samples from the unilateral ureteral obstruction (UUO) mice with or without being subjected to β-Mag treatment. (**A**) Summary of the in vivo experiment. The C57BL/6 mice underwent a UUO or sham operation on the first day. Subsequently, these mice were orally administered β-Mag (either 10 or 20 mg per kilogram of body weight daily). After drug therapy for 7 consecutive days, the kidney tissues of each mouse were harvested for histological and protein analyses. The grouping details are further described in the text. (**B**) The gross appearance, Hematoxylin and Eosin (H&E) staining, Masson trichrome stain for collagen I fiber, and immunohistochemical staining for α-smooth muscle actin (α-SMA), collagen I, and Vimentin in each study group. (**C**–**E**) Quantitative results of the histological findings in (**B**). Results are shown as % of protein expression of each experiment group. ** *p* < 0.01 when compared to the sham group; # *p* < 0.05 when compared to the UUO group.

A total of twenty mice were randomly assigned to five groups (N = 4 for each group): (1) a sham group, wherein the mice underwent isoflurane anesthesia and operation procedures without ureteral ligation and were orally fed normal saline for 10 days; (2) a UUO group, wherein the mice underwent isoflurane anesthesia, abdominal skin incision, muscle dissection, and ligation of the left ureter with 4–0 silk sutures on day 1 and were orally treated with normal saline for 7 days; (3) a UUO + β-Mag 10 mg/kg group, wherein the mice underwent left ureteral ligation on day 1 and were orally administered β-Mag (at 10 mg/kg of body weight) for 7 days; (4) a UUO + β-Mag 20 mg/kg group, wherein the mice underwent left ureteral ligation on day 1 and were orally administered 20 mg/kg of body weight of β-Mag for 7 days; and (5) a β-Mag 20 mg/kg group, wherein the mice were orally administered 20 mg/kg of body weight of β-Mag without a surgical intervention. All the mice were sacrificed on day 7, and their kidneys were harvested for histologic studies, and protein quantification expression analysis (Figure 1A). This animal study was approved by the Institutional Animal Care and Use Committee of Chung Shun Medical University (IACUC approval code: 2762).

### 2.3. Histopathological Assay

The mouse kidneys were removed, preserved in 10% formalin solution, and fixed in 10% formaldehyde for 24 h. The kidney tissues were embedded in paraffin, and a microtome was used to cut 3 μm thick sections, which were adhered to slides. Next, the slides were deparaffinized in xylene twice and then hydrated using a series of ethanol solutions with different concentrations. Finally, Hematoxylin and Eosin (H&E) and Masson’s trichrome staining were performed to analyze tissue morphology. An optical microscope operated at 200× magnification was used to make observations and capture images. Tubular injury was assessed by calculating the percentage of tubular atrophy and tubular cast formation using the following scoring system: Score 0: none; Score 1: <10%; Score 2: 10–25%; Score 3: 25–50%; Score 4: 50–74%; Score 5: >75%. At least 20 non-overlapping fields (magnification, ×200) per section for each kidney sample were examined. The percentage of positive staining area (brown color) for α-SMA and Vimentin was calculated using TissueFAXS Imaging Software 7.0 (TissueGnostics GmbH, Vienna, Austria) by dividing the positive area by the total area.

### 2.4. Cell Line and Culture Condition

The human kidney cell line HK2 (BCRC60097) is a proximal tubular epithelial cell obtained from normal kidney tissue. It was purchased from the Bioresource Collection and Research Center (BCRC, Taiwan) and cultured in Dulbecco’s Modified Eagle Medium/Nutrient Mixture F-12 (DMEM/F12), including 10% FBS, 1% nonessential amino acids (NEAA), 100 U/mL of penicillin–streptomycin, and 1 mM of sodium pyruvate. The cells were incubated at 37 °C in a sterile incubator with 5% CO_2_ and passaged when they reached 80–90% confluence.

### 2.5. Cell Growth Was Determined by MTT Assay

The HK-2 cells (3 × 10^4^ cells per well) were seeded into a 24-well cell culture plate overnight. On the next day, the cells were treated with different concentrations of β-Mag or TGF-β1 combined with β-Mag for 24 h. The culture medium was then removed, and 200 μL of MTT (0.5 mg/mL) was added into the 24-well cell culture plate, which was incubated at 37 °C for 2 h. After formazan crystals had formed, 500 μL of isopropanol was added per well, and shaking was performed for 15 min to dissolve the purple crystals. The absorbance of each well was measured at 570 nm using an ELISA reader, and the results were compared with those for the control group to calculate the relative cell viability.

### 2.6. Wound Healing Activity

A wound healing assay can be used to observe cell migration rates. HK-2 cells (3.5 × 10^5^) were placed into a 6-well cell culture plate equipped with Culture-Insert 2 wells (ibidi GmbH, Gräfelfing, Germany). On the next day, when each well was fully confluent, the Culture-Insert was removed. A serum-free medium was added, and images of the cells at 0 h were captured to observe their morphologies. The HK2 cells were pretreated with the TGF-β1 for 2 h and then supplemented with different concentrations of β-Mag for another 22 h. Images depicting the cell motility rate were photographed and measured using a phase-contrast microscope at 0 and 24 h. The wound healing rate was measured using the following equation: wound healing (%) = (A_0_ − A_24_/A_0_) × 100. (A_0_: the original wound site at 0 h; A_24_: the wound site at 24 h).

### 2.7. Immunofluorescence Staining

Immunofluorescence assays were performed as per our previous reports [21] and employed to examine the distribution and expression levels of Vimentin, Snail, and N-cadherin in each experimental kidney group. The slides were permeabilized (with 0.5% Triton X-100) and fixed (using 4% paraformaldehyde) for 20 min. Each experimental kidney group was then incubated with a blocking buffer (5% BSA) for 1 h. Then, the specific primary antibodies (Supplementary Table S1) were added at 4 °C overnight. On the next day, the secondary antibodies were added for 1 h. Finally, the slides were mounted with DAPI in the medium for 10 min. The stained slides and tissue sections were then examined under an Axio Imager A2 system (ZEISS MicroImaging GmbH, Munich, Germany), and images were captured at 400× magnification.

### 2.8. Gene Transfection for JNK Plasmid

First, HK2 (2 × 10^5^) cells were cultured in 6 cm dishes overnight. The JNK plasmid (1 μg) was mixed with Turbofect reagent in a serum-free medium and incubated for 5 min. After 6 h, the medium was replaced with fresh serum-containing medium for 18 h; then, β-Mag was added for 24 h, and subsequent experiments were carried out accordingly.

### 2.9. Immunoblotting

TGFβ1, TGFβ1 + β-Mag, or TGFβ1 + β-Mag co-treated with kinase inhibitor in HK2 cells (5 × 10^5^ cells) were seeded in 6 cm culture dishes for 24 h. After the cells were trypsinized, the added cells were lysed in lysis buffer (50 mM Tris, pH 7.5, 0.5 M NaCl, 1.0 mM EDTA, 10% glycerol, 1 mM β-mercaptoethanol, and 1% Nonidet P-40) containing protease inhibitors, and the supernatant was collected via centrifugation at 12,000× *g* for 15 min at 4 °C. The protein lysates were subjected to heat-induced denaturation at 100 °C for 10 min and subsequently loaded onto a 10% SDS-PAGE gel for electrophoretic separation. They were then immediately transferred onto a PVDF membrane. The membrane was blocked with TBST solution containing 5% BSA to prevent non-specific binding. The membrane was then incubated with the specific primary antibodies overnight at 4 °C. Subsequently, the membranes were incubated with corresponding horseradish peroxidase-linked antibodies for 2 h at room temperature and washed twice with a washing buffer. Specific protein expression was determined using an Immobilon Western Chemiluminescent HRP Substrate (Millipore, Billerica, MA, USA). Protein imaging and quantification were performed using the Cytiva ImageQuant 800 system. Detailed information on the primary and secondary antibodies is shown in Supplementary Table S1.

### 2.10. Statistical Analysis

All values are presented as the mean ± standard error of the mean (SEM). Student’s *t*-test was used to analyze the significance of differences between two groups. One-way analysis of variance (ANOVA) followed by Tukey’s multiple comparison test was used for examining parametric data across multiple groups. A *p*-value of ≤0.05 or ≤0.01 was considered statistically significant in this study.

## 3. Results

### 3.1. β-Mag Administration Resulted in the Regression of Kidney Fibrosis in UUO Mice

As shown in Figure 1A, the experiment entailed the β-Mag treatment of a UUO mouse model. The kidneys were resected from all the study mice on day 7, and gross and microscopic features were examined (Figure 1B). Based on the results of Hematoxylin and Eosin (H&E) staining, β-Mag treatment led to a significant decrease in the percentage of tubulointerstitial injuries and a lesser degree of tubular atrophy induced by UUO. Quantitative results were also obtained (Figure 1C). In the results regarding Masson trichrome staining, β-Mag administration after UUO induction decreased the deposition of collagen fibers, which were stained in blue (Figure 1D). The results of immunohistochemical (IHC) staining revealed a reduction in the expression of α-SMA and collagen I in the β-Mag-treated UUO mice in comparison to the sham group (Figure 1E). These results demonstrate that β-Mag alleviated renal fibrosis in UUO mice.

### 3.2. β-Mag Administration Caused a Downregulation of Epithelial-to-Mesenchymal Markers and Fibrotic Markers in UUO Mice

**Figure 2:**
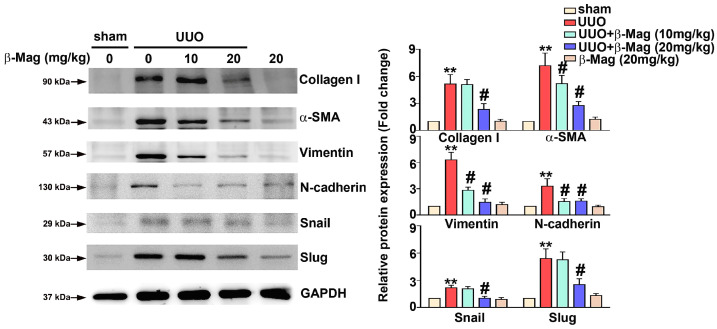
The inhibitory effect of β-Mag administration on the relative protein expression of EMT-associated markers and fibrosis-related markers in the unilateral ureteral obstruction (UUO) model. Western blot analysis regarding the protein expression of collagen I, α-SMA, Vimentin, N-cadherin, Snail, and Slug, for which glyceraldehyde-3-phosphate dehydrogenase (GAPDH) was used as a loading control. The histograms reveal the relative protein expression. Results are shown as relative fold change in protein expression in comparison to sham, where the expression level was set as 1. ** *p* < 0.01 when compared to the sham group; # *p* < 0.05 when compared to the UUO group.

The EMT is a very important process in renal fibrosis [22]. We evaluated the differential renal tissue expressions of collagen I, α-SMA, Vimentin, N-cadherin, Snail, and Slug proteins in all the no-treatment or treatment groups using immunoblotting. Compared to the sham operation group, the mice with a UUO showed significantly increased expression of EMT-associated proteins (Vimentin, N-cadherin, Snail, and Slug) and fibrotic proteins (collagen I, α-SMA). In contrast, the UUO mice orally administered β-Mag at a dose of 20 mg/kg per day for 7 days showed significant downregulation in all these proteins (Figure 2). The UUO mice that received β-Mag daily had reduced expression of EMT- and fibrosis-related markers.

### 3.3. Effect on Cell Growth of β-Mag Treatment with TGF-β1 in Cultured Human HK2 Cells

**Figure 3:**
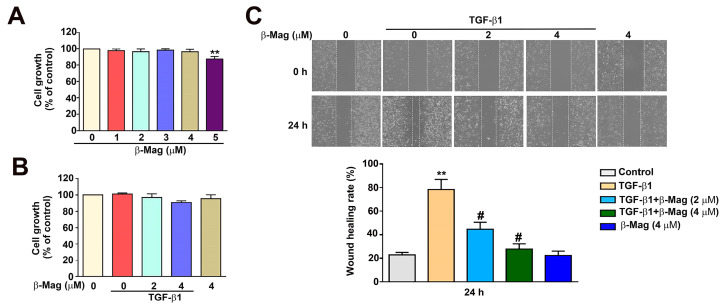
Effect of β-Mag treatment on TGF-β1-induced cell growth and motility in cultured human HK2 cells in vitro. (**A**) The results of the MTT assay, revealing the changes in cell proliferation after treatment with different concentrations of β-Mag. (**B**) Cell growth under the 24 h co-treatment with TGF-β1 and β-Mag at concentrations of 0, 2, and 4 µM. (**C**) The scratch wound healing assay demonstrated that β-Mag inhibited the cell migrating ability induced by TGF-β1. ** *p* < 0.01 when compared to the control group; # *p* < 0.05 when compared to the TGF-β1 group.

The MTT assay was utilized to assess the cell viability of human HK2 cells treated with various concentrations of β-Mag (1~5 μM) for 24 h. A β-Mag concentration of 5 µM was associated with significantly reduced cell survival and induced cell toxicity (Figure 3A). Co-treatment with β-Mag at concentrations of 2 and 4 µM and transforming growth factor-beta 1 (TGF-β1) did not inhibit cell viability when compared to the control (without β-Mag or TGF-β1 treatment) (Figure 3B). These β-Mag concentrations combined with TGF-β1 were used in the wound healing assay. The addition of β-Mag (2 and 4 µM) to the TGF-β1 treatment decreased the cell motility ability of HK2 cells at 24 h compared with TGF-β1 treatment alone (Figure 3C). These results suggest that β-Mag impaired the ability of TGF-β1 to induce cell motility under non-toxic conditions.

### 3.4. β-Mag Attenuated the Epithelial-to-Mesenchymal Transition and Fibrotic Markers Induced by TGF-β1 in Cultured Human HK2 Cells

**Figure 4:**
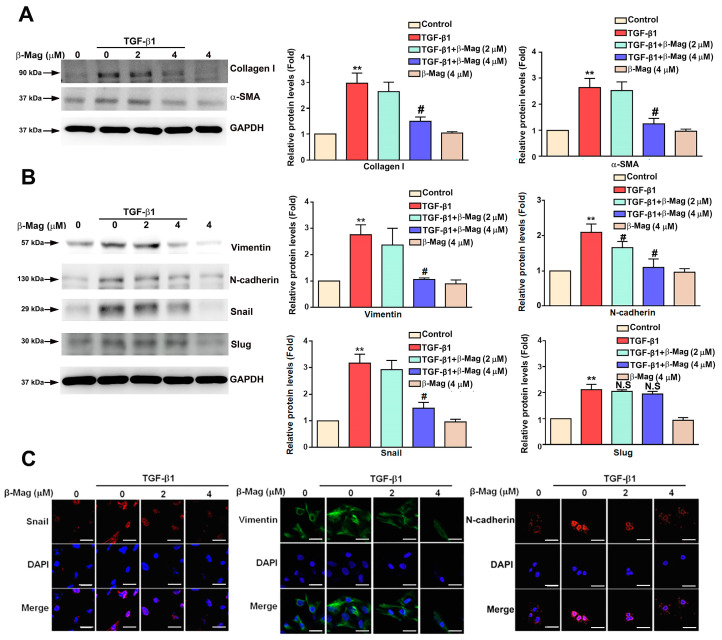
β-Mag inhibits TGF-β1-induced EMT-associated markers and fibrosis-related markers in HK2 cells. HK2 cells were co-treated with β-Mag (2 and 4 μM) and TGF-β1 for 24 h. (**A**) Western blot analysis comparing protein expression of collagen I and α-SMA between groups. (**B**) Western blot analysis comparing protein expression of Vimentin, N-cadherin, Snail, and Slug between groups. GAPDH was used as a loading control. (**C**) Immunofluorescence staining of Snail, Vimentin, and N-cadherin between treatment groups. Results are shown as relative fold change in protein expression in comparison to control, where the expression level was set as 1. ** *p* < 0.01 when compared to the control group; # *p* < 0.05 when compared to the TGF-β1 group. N.S, not significant. Scale bar: 20 μm.

Upon examining the role of the TGF-β1-induced EMT in the β-Mag-treated HK2 cells, it was observed that for the cultured HK2 cells, β-Mag co-therapy reduced the augmented protein expression of collagen I and α-SMA by TGF-β1 (Figure 4A). The expression of proteins indicative of mesenchymal transformation (Vimentin, N-cadherin, Snail) increased after the introduction of TGF-β1; it then declined under simultaneous β-Mag administration, but Slug expression was unaffected (Figure 4B). Similar results were found regarding immunofluorescence staining (Figure 4C). These results support the notion that β-Mag decreases the TGF-β1-induced EMT via targeting Vimentin/Snail/N-cadherin expression in HK2 cells.

### 3.5. β-Mag Diminished TGF-β1-Dependent Smad2 Phosphorylation-Mediated Snail/Vimentin in Cultured Human HK2 Cells

**Figure 5:**
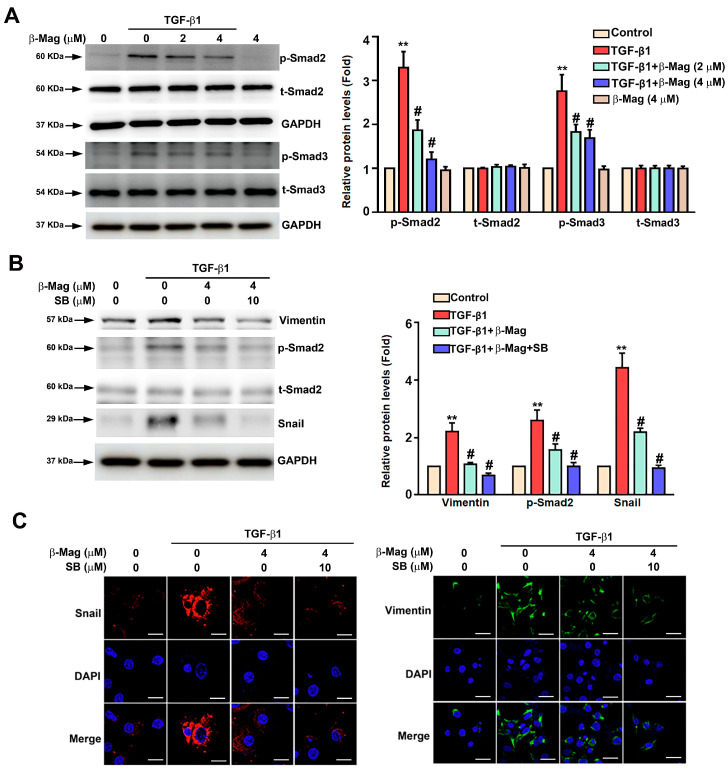
The Smad2/Smad3 pathway acting as a major target of β-Mag in the mediation of renal EMT. (**A**) HK2 cells were co-treated with β-Mag (2 and 4 μM) and TGF-β1 for 24 h. Then, Western blot analysis was conducted to demonstrate the expression of p-Smad2, p-Smad3, t-Smad2, and t-Smad3. (**B**) HK2 cells were added with or without SB431542 (SB, 10 μM), combined with or without TGF-β1, along with β-Mag (4 μM), and then the expression of p-Smad2, t-Smad2, Vimentin, and Snail was measured after β-Mag administration. GAPDH was used as a loading control. (**C**) Immunofluorescence staining of Snail and Vimentin between the treatment groups. Results are shown as relative fold change in protein expression in comparison to control, where the expression level was set as 1. ** *p* < 0.01 when compared to the control group; # *p* < 0.05 when compared to the TGF-β1 group. Scale bar: 20 μm.

Previous reports have suggested that the TGF-β1/Smad2/Smad3 signaling pathway is involved in EMT-mediated renal fibrosis [23]. In HK2 cells, TGF-β1 treatment enhanced Smad2 and Smad3 phosphorylation, a process inhibited by β-Mag co-treatment, for 24 h (Figure 5A). SB431542 is a Smad inhibitor of the TGF-β1/Smad signaling axis. The addition of SB431542 (SB; 10 µM) to β-Mag (4 µM) further decreased the phosphorylated Smad2 (p-Smad2) as well as Vimentin and Snail protein expression (Figure 5B). Results consistent with the immunofluorescence staining are shown in Figure 5C. 

### 3.6. β-Mag Downregulated TGF-β1-Induced p-JNK-Mediated p-Smad2 Targeting Snail/Vimentin Pathway

**Figure 6:**
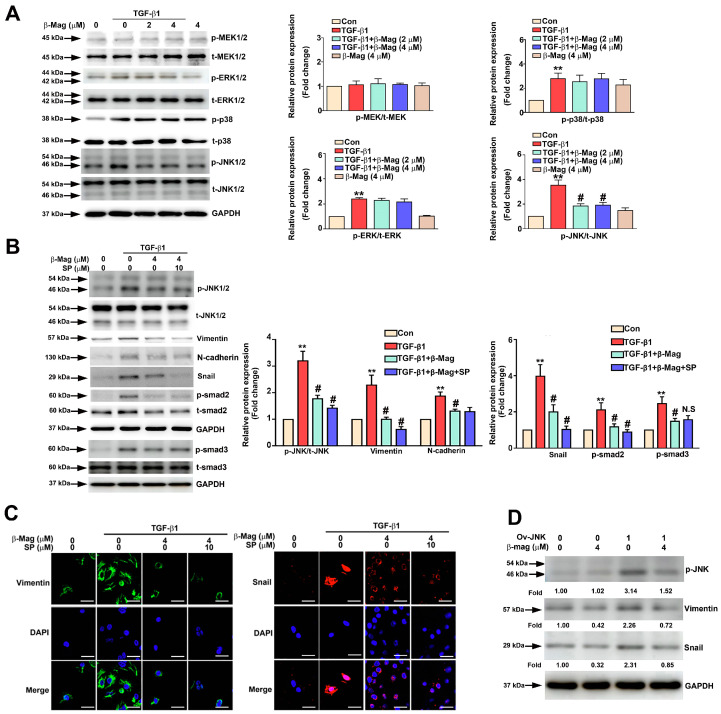
The effect of β-Mag on TGF-β1-induced JNK activation and the triggering of p-Smad2/Snail targeting Vimentin expression in HK2 cells. (**A**) HK2 cells were co-treated with β-Mag (2 and 4 μM) and TGF-β1 for 24 h. Then, using Western blot analysis, we determined the expression of MAPKs (p-MEK1/2, t-MEK1/2, p-ERK1/2, t-ERK1/2, p-p38, t-p38, p-JNK1/2, and t-JNK1/2). (**B**) HK2 cells were added with or without SP600125 (SP, 10 μM), combined with or without TGF-β1, along with β-Mag (4 μM), and the expression of p-JNK1/2, t-JNK1/2, p-Smad2, t-Smad2, p-Smad3, t-Smad3, Vimentin, N-cadherin, and Snail was measured using Western blotting. The histogram results are presented as the average fold change in the level of each protein normalized to GAPDH. (**C**) Immunofluorescence staining of Snail and Vimentin between the treatment groups. (**D**) After transfecting the JNK plasmid (Ov-JNK) in HK2 cells for 24 h, we added β-Mag for another 24 h and detected the protein expression of p-JNK1/2, Vimentin, and Snail. GAPDH was used as loading control. Results are shown as relative fold change in protein expression in comparison to control, where the expression level was set as 1. ** *p* < 0.01 when compared to the control group; # *p* < 0.05 when compared to the TGF-β1 group. N.S, not significant. Scale bar: 20 μm.

To elucidate the molecular mechanism underlying the antifibrotic effects of β-Mag against TGF-β1-induced JNK activation pathways, we examined the protein levels of MAPKs (MEK, ERK, p38, and JNK). TGF-β1 treatment enhanced p-JNK phosphorylation, which was decreased by co-treatment with β-Mag for 24 h, but there were no changes in the phosphorylation statuses of MEK/ERK or p38 kinase (Figure 6A). We sought to clarify the role of JNK activation in renal fibrosis. SP600125 (SP; 10 μM), a JNK inhibitor, significantly decreased the JNK phosphorylation, the expression of Vimentin and Snail, and the phosphorylated Smad2 (p-Smad2) protein levels in the TGF-β1 and β-Mag co-treatment in HK2 cells, but was not involved in phosphorylated Smad3 (p-Smad3) protein levels (Figure 6B). Results consistent with the immunofluorescence staining are shown in Figure 6C. In examining the role of JNK in the β-Mag-treated HK2 cells, using a transient transfection via an overexpressed JNK (Ov-JNK) assay, we found that β-Mag significantly decreased JNK-induced Snail and Vimentin expression in HK2 cells (Figure 6D). In summary, β-Mag hampered renal fibrosis through the inhibition of the JNK/Smad2-mediated Snail/Vimentin axis.

## 4. Discussion

**Figure 7:**
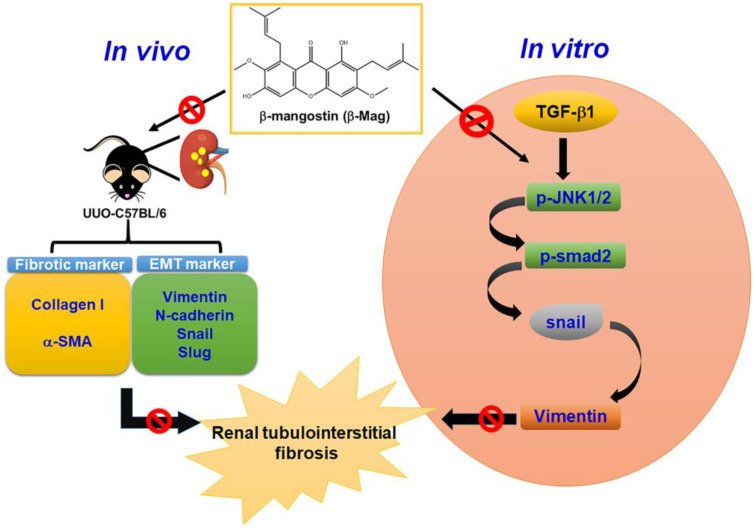
Summary of the proposed mechanism of action for β-Mag in suppressing renal tubulointerstitial fibrosis.

The primary findings of the current study are that β-Mag treatment resulted in diminished EMT features and a less severe degree of kidney fibrosis in the UUO model. The in vitro study on cultured HK-2 cells also revealed the effect of β-Mag on the alleviation of mesenchymal transformation and the expression of fibrotic molecules through the inhibition of Smad and JNK signaling. To the best of our knowledge, this is the first study that has examined and proved the in vitro and in vivo antifibrotic effects of inhibiting β-Mag to deter TGF-β1-induced JNK/Smad2-mediated Snail-dependent targeting of the Vimentin pathway in renal tubulointerstitial fibrosis (Figure 7).

Kidney fibrosis is characterized by the pathological alterations of each anatomic compartment in the kidney after different types of injury events. Glomerulosclerosis, tubulointerstitial fibrosis, vasculopathy, and perivascular fibrotic changes are important aspects of a fibrotic kidney [24]. The severity of the renal tubular atrophy and interstitial fibrosis serves as a predictor of the decline in the glomerular filtration rate as well as adverse renal outcomes [25,26,27]. A number of ongoing clinical trials are attempting to preserve kidney function by targeting the molecular pathways involved in renal fibrogenesis among patients with chronic kidney disease with various etiologies [6]. In our study, the results obtained using the in vivo UUO model proved that β-Mag could induce the regression of the tubular atrophy and fibrosis in the interstitium, a process that potentially has beneficial effects on kidney prognoses.

Although debate exists in this regard, the EMT is still regarded as one of the central drivers in the pathogenesis of kidney fibrosis, leading to a loss of the functional integrity of renal tubular epithelial cells as well as the progression of renal function decline [28,29]. During the EMT, these cells undergo cell cycle arrest and metabolic derangement, and the production of proinflammatory cytokines and leukocyte infiltration increase [5]. Recently, our previous research concluded that α-mangostin, another xanthone-containing compound identified in mangosteens, attenuated the renal EMT and the resultant renal fibrosis in UUO mice and in TGF-β1–induced fibrotic and EMT-related proteins administered to HK2 cells [30]. The current study also showed consistent results for β-Mag, but unlike β-Mag treatment, inhibiting TGF-β1 induced the JNK activation of Snail targeting Vimentin expression, not N-cadherin (Figure 6B). In summary, further investigation is required to determine whether the therapeutic blockade of the EMT contributes to the reversal of kidney fibrosis [22].

TGF-β1/Smad intracellular signaling is one of the important mediators of kidney fibrotic processes. The TGF-β1 molecule binds to the TGF-β1 receptor, which further leads to the phosphorylation of Smad2 and Smad3 [23]. After being phosphorylated, Smad2 and Smad3 combine with Smad4. The Smad2/Smad3/Smad4 complex shifts into the nucleus and alters the transcription of genes responsible for kidney fibrosis and inflammation [31,32]. The present investigation revealed that β-Mag reduced the TGF-β1-dependent phosphorylation of Smad2/Smad3 in vitro. Furthermore, β-Mag and the Smad inhibitor SB431542 additively inhibited the expression of the Snail protein, which indicated the role of Smad2 in alleviating EMT in cultured renal tubular epithelial cells. Indeed, the association between Smad2/3 upregulation and EMT in various tissues is well established based on prior studies [33]. However, in accordance with a rat study, Smad2 has also been observed to defend against Smad3-dependent collagen accumulation and renal fibrosis both in vivo and in vitro [34]. Because we did not measure the expression of other Smad proteins (such as Smad4) in our study, we could hardly tell whether the decrease in Smad2/3 activation after β-Mag treatment was secondary to β-Mag-induced antifibrotic effects. Further experiments are required to prove this idea.

The role of JNK signaling in triggering fibrogenesis in the kidneys and other organs has been previously investigated [10]. JNK activation enhanced the TGF-β-dependent, Smad4-independent production of fibronectin, one of the major components of the extracellular matrix [35]. Another in vitro study revealed that in human lung fibroblasts, JNK, activated by TGF-β1, increased the expression of connective tissue growth factor, a critical mediator of fibrosis [36]. Our study results are consistent with those obtained in a previous rat study in which it was disclosed that the JNK pathway was activated in a UUO model. However, a non-selective JNK-competitive inhibitor, CC-401, did reduce the infiltration of myofibroblasts and the accumulation of collagen IV fibers in the renal interstitium [37]. Consequently, targeting JNK-associated molecules could be a promising measure for treating or preventing acute and chronic kidney dysfunction [38]. Several studies have suggested that the JNK-mediated EMT pathway plays a crucial role in renal fibrosis. For example, Dr. Seo et al. found that Honokiol suppresses hepatic fibrosis and EMT by targeting the E-cadherin/GSK3β/JNK signaling axis [39]. Similarly, Dr. Li et al. demonstrated that Scutellarin inactivates the Smad/JNK pathways to suppress TGF-β1-induced epithelial fibrosis and EMT, thereby alleviating airway inflammation and remodeling in asthma [40]. Additionally, GA was shown to inhibit TGF-β1-induced tubular EMT in HK2 cells, partially by blocking both the TGF-β/Smad and JNK signaling pathways [41]. Another study reported that the JNK pathway plays an essential role in the PTX3-mediated promotion of renal fibrosis [42]. Based on these observations, it is implied that the JNK/EMT pathway is a critical factor in renal fibrosis. We utilized various EMT markers to assess the kidney fibrogenic pathways that are potentially mediated by β-Mag. A combination of in vivo and in vitro studies can bolster the findings. Such an experimental design provides solid evidence that β-Mag has the capability of alleviating renal fibrosis. Additionally, our first study found that JNK/Smad2 activation was involved in TGF-β1-triggered EMT-mediated renal fibrosis in β-Mag-treated HK2 cells.

Snail, an important transcription factor in renal embryonic development, is also highly expressed in an acute kidney injury (AKI) [43]. The literature indicates that Snail regulates several crucial biological processes associated with renal tubular interstitial fibrosis, including the mesenchymal reprogramming of tubular epithelial cells, the shutdown of fatty acid metabolism, cell cycle arrest, and inflammation in the microenvironment surrounding tubular epithelial cells [44], suggesting that Snail has the potential to serve as a therapeutic target in the progression of renal tubular interstitial fibrosis. In vitro and in vivo experiments show that Renalase inhibits renal fibrosis by suppressing the GSK-3β/Snail signaling pathway by reducing ER stress [45]. In a study by Sato et al., suppressing Smad3 activation during the TGF-β regulation of mechanical stress resulted in the induction of Snail inactivation and the blocking of epithelial phenotypic changes in tubular epithelial HK2 cells [46]. In this study, we found that the expression of JNK and Snail, a downstream effector of Smad2 in fibrosis, increased significantly in in vivo and in vitro models. When β-Mag was administered, renal fibrosis was reduced, and the expression of both Smad2 and Snail was decreased in the β-Mag treatment. After the overexpression of Snail, the antifibrotic effect of β-Mag was neutralized, indicating that β-Mag inhibited renal fibrosis by decreasing the expression of Snail and Vimentin. Therefore, our study is the first to discover that β-Mag regulates the Snail/Vimentin pathway, a finding that has not yet been confirmed by any other reports. However, how the transcription factor Snail regulates transcriptional activity and its direct or indirect binding to the Vimentin promoter in renal fibrosis remains to be further studied.

Currently, effective medical treatments for the retardation of chronic kidney disease (CKD) progression include the use of renin–angiotensin–aldosterone system blockers and sodium–glucose cotransporter 2 inhibitors. In addition to non-steroidal mineralocorticoid receptor antagonists, glucagon-like peptide-1 receptor agonists, used for select subpopulations with diabetic kidney disease, are the mainstay medical therapy for chronic kidney disease with the aim of improving long-term hard renal outcomes [47,48]. However, the prevalence and incidence of CKD and end-stage kidney disease keep rising worldwide. If we can discover a novel therapy that targets the upstream mechanisms associated with kidney disease progression, we may significantly alter the natural course of various kidney diseases. Antifibrotic therapy is a promising approach, and we are managing to find more molecules with the potential to treat kidney diseases. Thus, it is critical to conduct more clinical trials to prove the efficacy and safety of the clinical use of these natural compounds. Our study shows that β-Mag reversed renal EMT and exerted an antifibrotic effect in the earlier phases of kidney injury and subsequent repair. The combination of β-Mag with the standard treatment may have a synergistic impact on slowing kidney disease progression and decreasing the incidence of end-stage kidney failure. Further preclinical studies and clinical trials are required in order to prove the safety and efficacy of such treatments.

## 5. Conclusions

In conclusion, based on the in vivo and in vitro experiments, β-Mag retards the EMT and renal fibrosis by regulating the TGF-β/JNK/Smad2-mediated Snail/Vimentin axis.

## Data Availability

The authors will freely release all data supporting the published paper upon direct request to the corresponding author.

## Supplementary Materials

The following supporting information can be downloaded at https://www.mdpi.com/article/10.3390/cells13201701/s1: Table S1: Specific antibodies used in the Western blotting and immunofluorescence assay.
