# Supplementary material for: β-Mangostin Alleviates Renal Tubulointerstitial Fibrosis via the TGF-β1/JNK Signaling Pathway
Source: Cells. 2024 Oct 14;13(20):1701. doi: 10.3390/cells13201701 (PMC11505648; doi:10.3390/cells13201701)
Supplement: Supplementary file 1 [file cells-13-01701-s001.zip › cells-3212600-supplementary.pdf]

cells

MDPI

Article

β-Mangostin Alleviates Renal Tubulointerstitial Fibrosis via the TGF-β1/JNK Signaling Pathway

Po-Yu Huang [1,2,†], Ying-Hsu Juan [3,4,†], Tung-Wei Hung [5,6], Yuan-Pei Tsai [7], Yi-Hsuan Ting [7], Chu-Che Lee [8], Jen-Pi Tsai [2,9,*] and Yi-Hsien Hsieh [7,10,*]

1 Institute of Medical Sciences, Tzu Chi University, Hualien 970374, Taiwan; poyuhs13628@gmail.com
2 Division of Nephrology, Department of Internal Medicine, Dalin Tzu Chi Hospital, Buddhist Tzu Chi Medical Foundation, Chiayi 62247, Taiwan
3 Department of Chinese Medicine, Dalin Tzu Chi Hospital, Buddhist Tzu Chi Medical Foundation, Chiayi 62247, Taiwan; ddjjaa@tcts.seed.net.tw
4 School of Post-Baccalaureate Chinese Medicine, Tzu Chi University, Hualien 970374, Taiwan
5 School of Medicine, Chung Shan Medical University, Taichung 40201, Taiwan; a6152000@ms34.hinet.net
6 Division of Nephrology, Department of Medicine, Chung Shan Medical University Hospital, Taichung 40201, Taiwan
7 Institute of Medicine, Chung Shan Medical University, Taichung 40201, Taiwan; patty8782@gmail.com (Y.-P.T.); dys0090@gmail.com (Y.-H.T.)
8 Department of Medicine Research, Buddhist Dalin Tzu Chi Hospital, Chiayi 62247, Taiwan; dm731849@tzuchi.com.tw
9 School of Medicine, Tzu Chi University, Hualien 970374, Taiwan
10 Department of Medical Research, Chung Shan Medical University Hospital, Taichung 40201, Taiwan
* Correspondence: tsaininimd1491@gmail.com (J.-P.T.); hyhsien@csmu.edu.tw (Y.-H.H.)
† These authors contributed equally to this work.

Supplementary Materials

Citation: Huang, P.-Y.; Juan, Y.-H.; Hung, T.-W.; Tsai, Y.-P.; Ting, Y.-H.; Lee, C.-C.; Tsai, J.-P.; Hsieh, Y.-H. β-Mangostin Alleviates Renal Tubulointerstitial Fibrosis via the TGF-β1/JNK Signaling Pathway. *Cells* **2024**, *13*, x. https://doi.org/10.3390/cells13201701

Academic Editor(s): Prabhatchandra Dube

Received: 1 September 2024
Revised: 7 October 2024
Accepted: 11 October 2024
Published: 14 October 2024

Copyright: © 2024 by the authors. Submitted for possible open access publication under the terms and conditions of the Creative Commons Attribution (CC BY) license (https://creativecommons.org/licenses/by/4.0/).

Table S1. Specific antibodies used in the western blotting and immunofluorescence assay.

Western Blotting			
**Antibody**	**Dilution**	**Product Number**	**Company Source**
**Primary antibodies**			
Collagen I	1:1000	#72026	Cell Signaling Technology
α-SMA	1:1000	sc-32251	Santa Cruz Biotechnology
Vimentin	1:1000	#3634-100	BioVision
N-cadherin	1:1000	IR46-143	IReal Biotechnology
Snail	1:1000	#3895	Cell Signaling Technology
Slug	1:1000	sc-166476	Santa Cruz Biotechnology
p-Smad2	1:1000	#3108	Cell Signaling Technology
t-Smad2	1:1000	#5339	Cell Signaling Technology
p-Smad3	1:1000	#3108	Cell Signaling Technology
t-Smad3	1:1000	#9523	Cell Signaling Technology
p-MEK1/2	1:1000	sc-7995	Santa Cruz Biotechnology
t-MEK1/2	1:1000	sc-436	Santa Cruz Biotechnology
p-ERK1/2	1:1000	#9101	Cell Signaling Technology
t-ERK1/2	1:2000	#9102	Cell Signaling Technology
p-p38	1:1000	sc-166182	Santa Cruz Biotechnology
t-p38	1:1000	sc-7972	Santa Cruz Biotechnology
p-JNK1/2	1:1000	sc-6254	Santa Cruz Biotechnology
t-JNK1/2	1:1000	sc-7345	Santa Cruz Biotechnology
GAPDH	1:5000	#60004-1-Ig	Proteintech
**Secondary antibodies**			
anti-rabbit IgG	1:10000	AP132P	Merck Millipore
anti-mouse IgG	1:10000	AP124P	Merck Millipore
**Immunofluorescence**			
**Antibody**	**Dilution**	**Product Number**	**Company Source**
Vimentin	1:250	sc-32322	Santa Cruz Biotechnology
Snail	1:100	A5544	ABclonal
N-cadherin	1:100	A19083	ABclonal
